# BET inhibitors induce apoptosis through a MYC independent mechanism and synergise with CDK inhibitors to kill osteosarcoma cells

**DOI:** 10.1038/srep10120

**Published:** 2015-05-06

**Authors:** Emma K Baker, Scott Taylor, Ankita Gupte, Phillip P Sharp, Mannu Walia, Nicole C Walsh, Andrew CW Zannettino, Alistair M Chalk, Christopher J Burns, Carl R Walkley

**Affiliations:** 1 St. Vincent’s Institute, Fitzroy, 3065 VIC, Australia; 2 Department of Medicine, St. Vincent’s Hospital, University of Melbourne, Fitzroy, 3065 VIC, Australia; 3 The Walter and Eliza Hall Institute of Medical Research, Parkville, VIC, 3052, Australia; 4 Myeloma Research Laboratory, School of Medical Sciences, Faculty of Health Sciences, University of Adelaide, Adelaide, SA 5005, Australia and Cancer Theme, South Australian Health and Medical Research Institute, Adelaide, SA 5000, Australia; 5 The University of Melbourne, Department of Medical Biology, 3010 VIC, Australia; 6 ACRF Rational Drug Discovery Centre, St. Vincent’s Institute, Fitzroy, 3065 VIC, Australia

## Abstract

Osteosarcoma (OS) survival rates have plateaued in part due to a lack of new therapeutic options. Here we demonstrate that bromodomain inhibitors (BETi), JQ1, I-BET151, I-BET762, exert potent anti-tumour activity against primary and established OS cell lines, mediated by inhibition of BRD4. Strikingly, unlike previous observations in long-term established human OS cell lines, the antiproliferative activity of JQ1 in primary OS cells was driven by the induction of apoptosis, not cell cycle arrest. In further contrast, JQ1 activity in OS was mediated independently of *MYC* downregulation. We identified that JQ1 suppresses the transcription factor *FOSL1* by displacement of BRD4 from its locus. Loss of *FOSL1* phenocopied the antiproliferative effects of JQ1, identifying FOSL1 suppression as a potential novel therapeutic approach for OS. As a monotherapy JQ1 demonstrated significant anti-tumour activity *in vivo* in an OS graft model. Further, combinatorial treatment approaches showed that JQ1 increased the sensitivity of OS cells to doxorubicin and induced potent synergistic activity when rationally combined with CDK inhibitors. The greater level of activity achieved with the combination of BETi with CDK inhibitors demonstrates the efficacy of this combination therapy. Taken together, our studies show that BET inhibitors are a promising new therapeutic for OS.

Osteosarcoma (OS) is the most common primary tumour of bone and predominantly affects children and adolescents. OS is a heterogeneous disease characterised by atypical osteoblast differentiation and production of abnormal osteoid. The most common diagnosis, conventional OS, presents as one of three subtypes; osteoblastic, fibroblastic and chondroblastic^1^. OS has relatively poor patient outcomes particularly in cases presenting with metastases or recurrent disease. Survival rates of 60–70% are achievable if patients have localised tumours^2^. At diagnosis, 25% of initial diagnoses and the majority of patients with recurrent disease have metastasis^3^. These patients have only ~30% chance of survival^2^. Contrasting with the rapid gains in our understanding of the genetics and cell biology of OS, there have been few new treatments introduced in the last three decades. OS is managed with multi-agent chemotherapy combined with surgical resection and treatment-related morbidity is common for OS patients^4^. New targeted therapies are urgently required to increase the efficacy of current therapy and reduce the risk of long-term therapy-related side effects.

The recurrent genetic lesions in OS are being rapidly uncovered. Most notable are loss of function mutations of *TP53* in essentially all OS and recurrent mutations in *RB, ATRX* and *DLG2*^5^,^6^,^7^. In addition to loss of function mutations, amplification and overexpression of *MYC* and *RUNX2* has been linked with OS pathogenesis including disease development, chemotherapy resistance, metastatic potential, poor response and inferior outcomes^8^,^9^,^10^,^11^,^12^,^13^,^14^. The potential therapeutic utility of targeting MYC in OS has been demonstrated in a conditional murine transgenic model, where transient *Myc* suppression induced OS regression^15^. Increased expression of *FOS* has been demonstrated in OS^16^. Mice lacking *Fos* had an osteopetrotic phenotype^17^, and reciprocally, transgenic mice over-expressing *Fos* developed OS^18^ indicating that Fos plays a role in OS pathogenesis. A closely related Fos family member, Fosl1, can rescue the bone phenotype of mice lacking Fos indicating a degree of functional redundancy^19^. Loss- and gain-of-function models indicate that Fosl1 acts as a positive regulator of bone formation^20^,^21^. Despite some redundancy in their roles, only Fos over-expression resulted in OS^21^. Collectively these studies suggest targeted inhibition of *MYC*, *RUNX2, FOS* or potentially *FOSL1* may represent a novel therapeutic approach for OS.

Pharmacological inhibitors of the bromodomain and extra terminal domain (BET) protein family, including JQ1, I-BET151 and I-BET762, demonstrate anti-tumour activity in a range of malignancies^22^,^23^,^24^,^25^,^26^,^27^,^28^,^29^,^30^. BET inhibitors (BETi) bind the acetylation recognition pocket of the BET proteins, displacing them from chromatin^24^,^26^. BRD4 inhibition in particular has been linked with the antiproliferative responses, and drives the disruption of oncogenic pathways^25^,^26^,^27^. In many models, transcriptional suppression of *MYC* is proposed as the primary mechanism of BETi action^24^,^25^,^28^,^30^. However recent studies demonstrate BETi can exert antiproliferative activity through suppression of alternative gene targets^27^,^29^. In lung cancer, the effects of JQ1 were attributed to inhibition of a network of FOS related genes including *FOSL1*^27^. As a number of the targets of BETi are involved in OS, we tested if BETi showed therapeutic activity against OS using a panel of primary OS cell cultures to provide a closer representation of the *in vivo* tumour.

Here we show that primary OS cell cultures derived from different OS models (murine, human), subtypes and primary vs metastatic sites are highly sensitive to BETi. JQ1 antiproliferative effects in primary OS derived cells were mediated by rapid induction in apoptosis, yet primary normal osteoblasts were protected from the pro-apoptotic effects. We show *FOSL1* is a direct target of BRD4 in OS and JQ1 suppresses *FOSL1* transcription, independent of *MYC* suppression. Furthermore we provide proof-of-principle that JQ1 can enhance standard OS treatments and be combined with CDK inhibitors to synergistically kill OS cells. Collectively these findings highlight the therapeutic potential of using BETi alone or in combination to treat OS.

## Results

### OS cells are sensitive to BET inhibition

**Figure 1:**
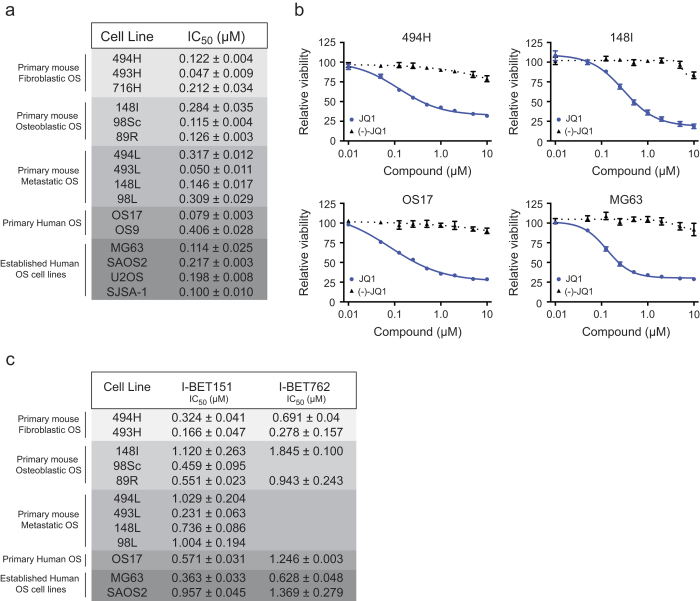
BET inhibitors have potent activity against OS cell cultures (**a**) IC_50_ values of OS cell cultures treated with JQ1 for 72 hrs. Mean IC_50_ values ± SEM (n = 2-4). (**b**) Dose response curves of mouse (494H, primary mouse fibroblastic; 148I, primary mouse osteoblastic) and human (OS17, primary human; MG63, established human OS cell line) cells treated with JQ1 or (-)-JQ1 for 72 hrs. Mean cell viability ± SEM (n = 3-4). (**c**) IC_50_ values of cells treated with I-BET151 and I-BET762 for 72 hrs. Mean IC_50_ values ± SEM (n = 2).

We treated a panel of genetically diverse human and mouse OS cultures, including long term established human OS cell lines and low passage primary cell cultures derived from genetically engineered mouse models (GEMM) of OS or human xenograft derived material^31^,^32^, with the BETi (+)-JQ1 (JQ1). The murine OS cultures were derived from two GEMM of human OS that recapitulate the OS fibroblastic and osteoblastic subtypes^33^,^34^. Primary tumour derived cultures were used to provide a closer representation of the *in vivo* tumour by limiting potential changes related to long term *in vitro* culture. JQ1 showed antiproliferative activity in all OS cells assessed at 72 hrs (Fig. 1a). An IC_50_ concentration in the low nanomolar range (0.047 μM to 0.406 μM) was observed across subtypes and species (Fig. 1a). No appreciable differences in IC_50_ were apparent if cell lines were clustered based on species, histological subtype, tumour site (primary vs metastasis), time in culture, or basal proliferation rate (Fig. 1a and Supplementary, Fig. 1). To demonstrate specificity, we tested the inactive (-)-JQ1 enantiomer that does not inhibit BET bromodomains^26^. (-)-JQ1 showed no appreciable effects on OS cells at concentrations up to 10 μM (Fig. 1b). Additional testing was undertaken with two structurally unrelated BETi compounds, I-BET762 and I-BET151. Both compounds demonstrated antiproliferative activity in all cultures examined at 72 hrs, with IC_50_ values predominantly in the nanomolar range (Fig. 1c), revealing a potent class effect of BETi on OS cells.

### BET inhibition induces apoptosis in primary OS

**Figure 2:**
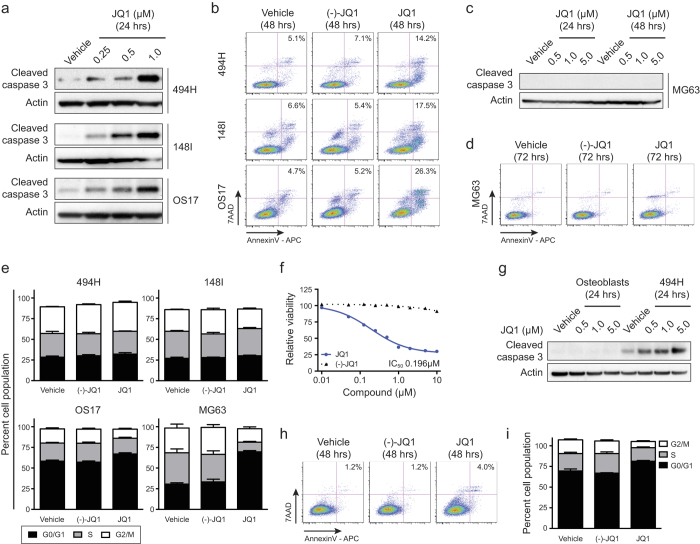
JQ1 induces apoptosis and cell cycle arrest in OS cell cultures (**a**) Induction of cleaved caspase 3 in primary OS cell cultures (494H, primary mouse fibroblastic; 148I, primary mouse osteoblastic; OS17, primary human) treated with JQ1 for 24 hrs. (**b**) Annexin V and 7AAD staining of OS cells treated with 1 μM JQ1, 1 μM (-)-JQ1 or vehicle (DMSO) for 48 hrs. Plots are representative biological experiments with mean number of Annexin V positive cells (n = 3). (**c**) Cleaved caspase-3 levels in MG63 cells (established human OS cell line) treated with JQ1 for 24–48 hrs. (**d**) Annexin-V/7AAD staining of MG63 cells treated with 1 μM JQ1, 1 μM (-)-JQ1 or vehicle (DMSO) for 72 hrs. Plots are representative biological experiments with mean number of Annexin-V positive cells (n = 3). (**e**) Cell cycle profile of OS cells treated for 24 hrs with 1 μM JQ1, 1 μM (-)-JQ1 or vehicle (DMSO). Mean cell population percentage ± SEM (n = 4). (**f**) Dose response curves of mouse normal osteoblast cells treated with JQ1 or (-)-JQ1 for 72 hrs. Mean cell viability ± SEM (n = 3). (**g**) Cleaved caspase-3 levels in mouse normal osteoblast cells and mouse 494H cells treated with JQ1 for 24 hrs. (**h**) Annexin-V/ 7AAD staining of mouse normal osteoblast cells treated with 1 μM JQ1, 1 μM (-)-JQ1 or vehicle (DMSO) for 48 hrs. Plots are representative biological experiments with mean number of Annexin-V positive cells (n = 3).

To identify the cellular mechanism underlying the antiproliferative effects of JQ1 in OS we assessed apoptosis and cell cycle status in representative primary OS cell cultures (494H, primary mouse fibroblastic; 148I, primary mouse osteoblastic; OS17, primary human). JQ1 rapidly induced apoptosis in a dose-responsive manner, evidenced by increased levels of cleaved caspase-3 within 24 hrs (Fig. 2a). A significant increase in cells that were Annexin-V positive compared to DMSO or (-)-JQ1 treatments in primary OS cell cultures was evident following treatment with 1 μM JQ1 for 48 hrs (Fig. 2b). Interestingly, the long established MG63 cell line, despite exhibiting an equally pronounced antiproliferative response to JQ1 (Fig. 1a,b), did not undergo apoptosis after extended treatment times (48–72 hrs) (Fig. 2c,d). To further investigate the antiproliferative effects, we assessed if JQ1 altered the cell cycle distribution of the cells. The mouse OS cultures (494H, 148I) were largely unaffected by treatment with 1 μM JQ1 for 24 hrs (Fig. 2e). Only a marginal decrease in the proportion of cells in the S and G_2_/M phases with a concurrent increase in cells in G_0_/G_1_ were observed in the human OS17 primary cell culture (Fig. 2e). In contrast, the established MG63 cell line showed an accumulation of cells in G_0_/G_1_, indicative of a cell cycle arrest.

We next examined the specificity of the anti-OS activity of JQ1 by assessing its effects on normal wild-type primary osteoblast cultures. JQ1 treatment for 72 hrs suppressed the proliferation of primary osteoblast cultures at an IC_50_ value of 0.196 μM (Fig. 2f) but did not reduce cell viability at time points when apoptosis was induced in JQ1 treated primary OS cultures (24–48 hrs) (Fig. 2g,h). A small decrease in the proportion of osteoblast cells in the S and G_2_/M phases with a concurrent increase in cells in G_0_/G_1_ were observed following 24 hrs of JQ1 treatment (Fig. 2i). Taken together, these data demonstrate that JQ1 exerts an antiproliferative activity against primary OS cell cultures by inducing apoptosis rather than preventing cell cycle progression. Importantly normal osteoblasts are protected from the pro-apoptotic effects of JQ1.

### JQ1 exhibits *in vivo* activity

**Figure 3:**
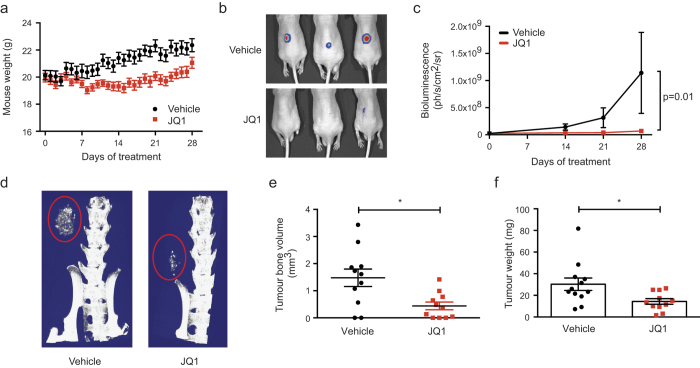
JQ1 OS activity *in vivo* Subcutaneous graft model of OS with 494H fibroblastic OS cells. (**a**) Body weight of mice undergoing treatment with JQ1 (50 mg/kg) or vehicle for 28 days. Mean weight ± SEM (n = 12). (**b**) Representative whole body bioluminescence images of mice at 28 days post treatment with JQ1 or vehicle. (**c**) Bioluminescence imaging assay of tumour burden in mice at day 0, 14, 21 and 28 post treatment with JQ1 or vehicle. Mean bioluminescence ± SEM (n = 12). Data was log transformed to normalise variances before analysis by 3-way ANOVA. (**d**) Representative microCT reconstructed images of tumours in mice treated for 28 days with JQ1 or vehicle. Tumour mass is circled in red. (**e**) MicroCT analysis of tumour bone volume in mice treated for 28 days with JQ1 or vehicle. Mean tumour bone volume ±SEM (n = 12). * *p* < 0.05 Mann Whitney ranks test. (**f**) Tumour weights from mice treated with JQ1 or vehicle for 28 days. Data is presented as mean ± SEM (n = 12, from two separate studies). * *p* < 0.05 calculated using Mann Whitney ranks test.

To investigate the *in vivo* therapeutic effect of JQ1 we utilised a luciferase tagged subcutaneous graft model. This model paralleled that used by the Pediatric Preclinical Testing Program for testing solid tumours^32^,^35^. This approach enabled an assessment of the OS intrinsic effects of the drug, independent of other cell types such as osteoclasts. The 494H fibroblastic OS culture was selected for the model as its JQ1 sensitivity and biological response profile were representative of other primary OS cell cultures tested. Mice were injected with OS cells subcutaneously on the dorsal hind flank. The tumour was allowed to establish for 1 week prior to mice receiving daily treatments with JQ1 (50 mg/kg) or vehicle for 28 days. Mice in both groups demonstrated normal eating and behavioural responses, although a lag in weight gain was observed in the JQ1 treatment group compared to vehicle controls (Fig. 3a). There was a striking suppression of proliferation of the OS in JQ1 treated mice (Fig. 3b,c). MicroCT analysis at day 28 showed that tumour bone volume was reduced on average by 70% in JQ1 treated mice (Fig. 3d,e). Tumour masses were on average 50% smaller than vehicle treated mice (Fig. 3f). Taken together, these data demonstrate JQ1 has potent anti-OS activity *in vivo*.

### BRD4 knockdown phenocopies BET inhibitors in OS

**Figure 4:**
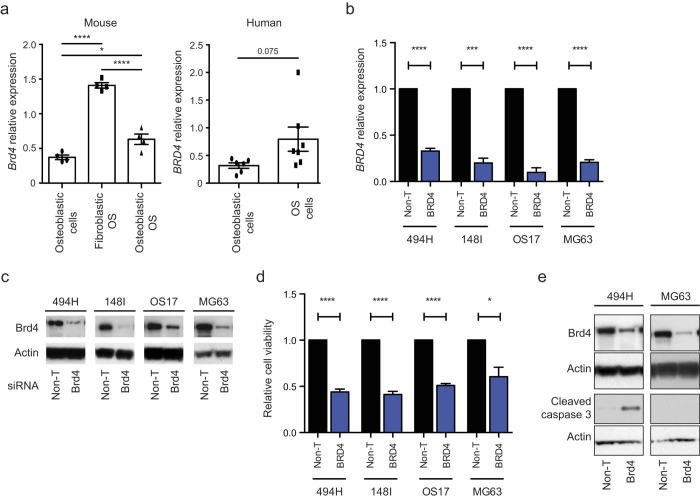
BRD4 knockdown phenocopies BET inhibitor activity in OS cells (**a**) *BRD4* transcript levels in mouse primary fibroblastic OS (494H, 493H, 716H, 202 V), mouse primary osteoblastic OS (148I, 98Sc, 89R, 147H) and human OS (U2OS, SJSA-1, B143, MG63, SAOS-2, G292, OS17) cells compared to normal osteoblast cells (n = 4-6 independently derived samples) quantified by QPCR. Mean relative expression ± SEM. *p < 0.05, ****p < 0.0001 ANOVA Tukey multiple comparisons test and student’s t test. (**b**-**d**) Human and mouse OS cells were transfected with siRNAs targeting BRD4 or a pool of non-targeting siRNAs (Non-T) and effects on *BRD4 *mRNA levels, protein expression and cell viability were assessed. (b) *BRD4* transcript levels relative to Non-T cells quantified by QPCR. Mean fold change ± SEM (n = 3). ***p < 0.001, ****p < 0.0001 student’s t test.. (**c**) Western blot analyses showing BRD4 protein levels in siRNA transfected OS cells. (**d**) Cell viability in siRNA transfected OS cells. Cell viability levels are relative to Non-T cells. Mean fold change ± SEM (n = 3). *p < 0.05, ****p < 0.0001 student’s t test. (**e**) Western blot analyses of Brd4 and cleaved caspase 3 levels in 494H cells (primary mouse fibroblastic) or MG63 cells (established human OS cell line) transfected with siRNAs targeting BRD4 or a pool of non-targeting siRNAs (Non-T) for 72 hrs.

To assess the contribution of the individual BET isoforms, we first determined that *Brd2*, *Brd3 and Brd4* are expressed in mouse OS cells (Supplementary Fig. 2a). Similarly, evaluation of microarray data demonstrated that *BRD2*, *BRD3* and *BRD4* are also expressed in human OS (Supplementary Fig. 2b). We confirmed the expression of *BRD2*, *BRD3* and *BRD4* in our human and mouse OS cells compared to normal primary osteoblastic cells using QPCR. There was increased relative expression of *Brd4, Brd2 and Brd3* in murine OS, with the highest levels in fibroblastic OS cells compared to normal osteoblastic cells (Fig. 4a and Supplementary Fig. 3a-b). *BRD4* expression was only moderately elevated in human OS cells compared to normal human osteoblasts (Fig. 4a and Supplementary Fig. 3c-d).

BRD4 inhibition has been strongly implicated in mediating the activity of JQ1^25^,^27^. To determine the contribution of BRD4 inhibition, we used siRNA against *BRD4* in mouse and human OS. Reducing BRD4 levels (Fig. 4b,c) led to almost a 50% reduction in cell viability in all OS cultures examined (Fig. 4d). The anti-proliferative effect in primary OS cells (494H) was due to the induction of apoptosis, evidenced by increased levels of cleaved caspase-3 (Fig. 4e). Interestingly, consistent with the response to JQ1, the anti-proliferative response to BRD4 loss in MG63 cells was not due to induction of apoptosis (Fig. 4e). JQ1 also inhibits the activities of BRD2 and BRD3. siRNA targeting *Brd2* and *Brd3* in mouse OS cells also reduced cell viability to some extent (10-30%) (Supplementary Fig. 4). Therefore BRD4 inhibition is the primary mediator of JQ1 activity in OS, but cumulative effects from the inhibition of all three BET proteins may contribute.

### JQ1 suppresses FOSL1, independent of MYC inhibition

**Figure 5:**
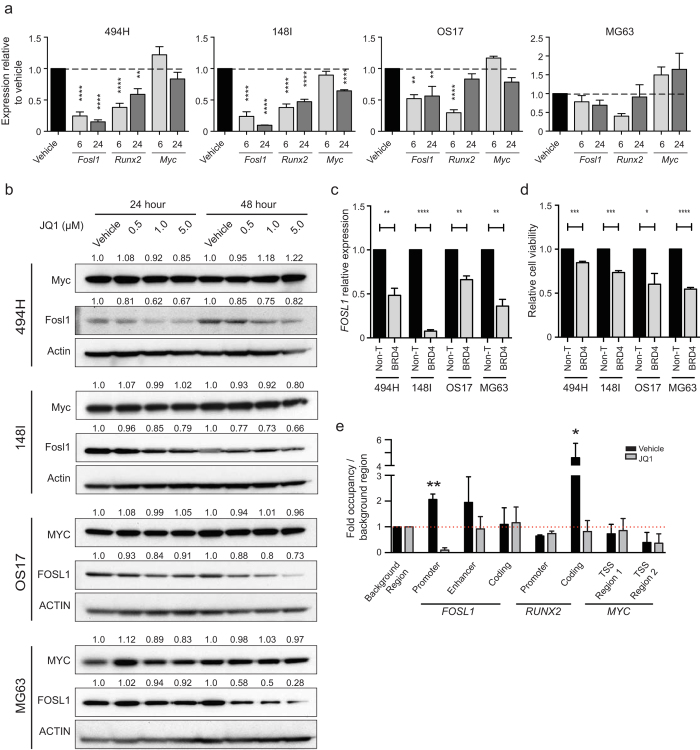
BET inhibitor activity in OS cells is driven by *>FOSL1* suppression, independent of *MYC* downregulation (**a**) Transcript levels of *MYC*, *FOSL1 and RUNX2* were evaluated by QPCR in OS cells treated for 6 hrs or 24 hrs with 500 nM JQ1 or vehicle (DMSO). Mean fold change ± SEM (n = 3). **p < 0.01, ****p < 0.0001 ANOVA Dunnett multiple comparisons test. (**b**) Western blot analyses showing MYC and FOSL1 protein levels in OS cell cultures treated with JQ1 or vehicle (DMSO) for 24–48 hrs. Protein band intensities quantified by ImageJ. (**c**) *FOSL1* transcript levels quantified by QPCR in OS cells transfected with siRNAs targeting *FOSL1* or a pool of non-targeting siRNAs (Non-T) for 72 hrs. Mean fold change ± SEM (n = 3). **p < 0.01, ****p < 0.0001 student’s t test. (**d**) Cell viability in OS cells transfected with siRNAs targeting *FOSL1* or a pool of non-targeting siRNAs (Non-T) for 72 hrs. Mean fold change ± SEM (n = 3). *p < 0.05, ***p < 0.001, ****p < 0.0001 student’s t test. (**e**) ChIP analysis of BRD4 enrichment at *FOSL1*, *RUNX2* and *MYC* loci in human OS17 cells treated with 1 μM JQ1 or vehicle (DMSO) for 24 hrs. Enrichment is normalised to the total input DNA and a background region devoid of genes. Mean ± SEM (n = 3). * *p* < 0.05, ** *p* < 0.01 students *t*-test.

To define the molecular mechanisms of BETi activity in OS, we examined genes transcriptionally downregulated by JQ1 in other cancers^25^,^27^. We focused on *MYC*, *FOSL1* and *RUNX2* as potential targets due to their link with OS or osteoblast biology. Firstly, we examined expression changes in OS cultures after JQ1 treatment (500 nM) for 6-24 hrs to identify early effects of JQ1. In contrast to many reports of *MYC* suppression in response to JQ1^23^,^25^,^28^,^29^,^30^,^36^, we saw either no reduction or, in some instances, slightly increased *MYC* expression following 6 hrs of treatment (Fig. 5a). After 24 hrs, *MYC* expression had only moderately reduced in 148I cells. Treatment with increasing concentrations of JQ1 over 24–48 hrs had negligible effect on MYC protein levels in all OS cells examined (Fig. 5b). Hence, under identical conditions to those that induced apoptosis or cell cycle arrest, we failed to observe changes in *MYC* expression suggesting MYC inhibition is not mediating the effects of JQ1 in OS.

In contrast to *MYC* expression, robust reductions in *FOSL1* and *RUNX2* levels occurred within 6 hrs of treatment (Fig. 5a). *RUNX2* expression was decreased at 6 hrs, but expression in human OS cells was effectively restored after 24 hrs (Fig. 4a). *FOSL1* expression levels were significantly repressed within 6 hrs of JQ1 treatment, except the MG63 cell line, which only showed a marginal reduction in expression at 24 hrs (Fig. 5a). Correspondingly, FOSL1 protein levels decreased with JQ1 treatment (Fig. 5b) suggesting FOSL1 could be a direct target of BETi.

To determine whether FOSL1 suppression may mediate the response to JQ1, we used siRNA to *FOSL1*. Knockdown of *FOSL1* was confirmed (Fig. 5c), and significantly reduced viability up to 45% compared to non-targeting siRNAs (Fig. 5d). While the reduction in cell viability was less than observed with either *BRD4* knockdown or JQ1 treatment, the results support a reduction in FOSL1 mediating the effects of JQ1.

The rapid reductions of *FOSL1* in response to JQ1 suggested JQ1 may be directly displacing BRD4 from the *FOSL1* locus. We performed chromatin immunoprecipitation to map BRD4 occupancy at the *FOSL1* locus. BRD4 was localised at the *FOSL1* promoter and enhancer regions (Fig. 5e). Following JQ1 treatment, BRD4 was displaced, most prominently from the promoter. We observed minimal enrichment of BRD4 at the *RUNX2* promoter region and its occupancy was unchanged in response to JQ1 treatment. BRD4 was localised in the coding region of *RUNX2*, and was displaced following JQ1 treatment (Fig. 5e). Enrichment of BRD4 at known sites surrounding the *MYC* transcription start site in OS cells was weak and occupancy was not reduced following JQ1 treatment, consistent with a lack of MYC transcriptional response to JQ1 treatment. These results define *FOSL1* as a direct target of BRD4 in OS cells.

### JQ1 enhances standard OS chemotherapy and synergistically combines with CDK inhibitors

**Figure 6:**
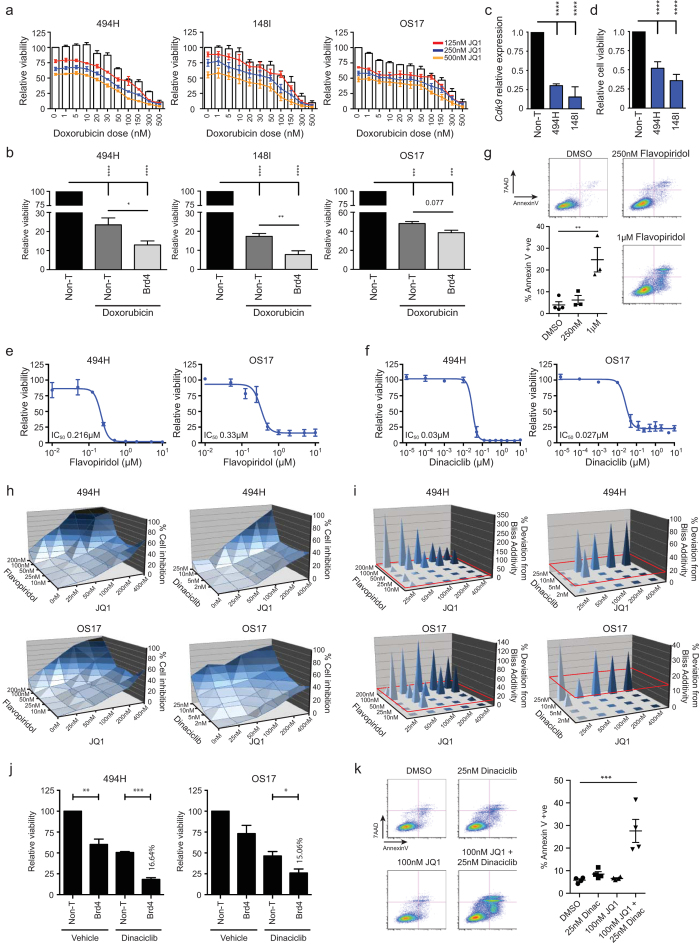
JQ1 enhances standard OS chemotherapy and synergistically combines with CDK inhibitors (**a**) Dose response of OS cells treated with doxorubicin in the absence (columns) or presence (lines) of JQ1 for 72 hrs. Mean viability ± SEM (n = 3). (**b**) OS cells transfected with BRD4 siRNA or non-targeting siRNA (Non-T) and treated with 100 nM doxorubicin or vehicle (DMSO). Mean fold change cell viability ± SEM, (n = 3). ANOVA Tukey multiple comparisons test. (**c**) *Cdk9* mRNA levels in mouse OS cells transfected with *Cdk9* siRNA or Non-T siRNAs (Non-T) for 72 hrs. Mean fold change expression ± SEM (n = 3). ANOVA Dunnett multiple comparisons test. (**d**) Cell viability in mouse OS cells transfected with *Cdk9* siRNA or Non-T siRNAs for 72 hrs. Mean fold change viability ± SEM (n = 3). ANOVA Dunnett multiple comparisons test. (**e-f**) Dose response curves of cells treated with flavopiridol or dinaciclib for 48 hrs. Mean ± SEM (n = 2). (**g**) AnnexinV/7AAD staining of OS17 cells treated with flavopiridol or vehicle (DMSO) for 24 hrs. Representative FACS plots; mean ± SEM (n = 4 DMSO, n = 3 Flavopiridol treated). ANOVA Dunnett multiple comparisons test. (**h**) OS cells treated with combinations of JQ1 with flavopiridol or dinaciclib for 72 hrs, represented as mean viability inhibition (n = 3). (**i**) Synergy effects of treatment with JQ1 combined with flavopiridol or dinaciclib for 72 hrs. Bliss additivity deviations greater than 15% were considered synergistic (red baseline), (n = 3). (**j**) Cell viability and synergy in OS cells transfected with BRD4 siRNA or Non-T siRNA and treated with 25 nM dinaciclib or vehicle (DMSO) for 72 hrs. Represented as mean cell viability normalised to vehicle treated Non-T siRNA ± SEM, (n = 3). Synergy is represented as mean percent deviation from a predicted additive response (Bliss additivity) (n = 3). (**k**) AnnexinV/7AAD staining of OS17 cells treated with combinations of JQ1 and dinaciclib for 48 hrs. Plots are representative biological experiments; results plotted as mean ± SEM Annexin-V positive cells (n = 4 per group). ANOVA Dunnett multiple comparisons test. For all panels: *p < 0.05, **p < 0.01, ***p < 0.001, ****p < 0.0001.

Chemotherapy treatment has improved the outcome of OS patients with localised tumours, yet therapy-related morbidity and poor response in patients with metastatic disease still remain significant hurdles^2^,^4^. We explored whether the addition of JQ1 to standard therapy could increase the efficacy of treatment *in vitro*. We treated a panel of primary OS cells (494H, 148I, OS17) with a dose range of doxorubicin combined with low dose JQ1 for 72 hrs and monitored cell proliferation responses. The addition of JQ1 was additive to the cytotoxic effects of doxorubicin suggesting JQ1 can chemo-sensitise OS cells (Fig. 6a). To further explore the role of BETi in OS response to chemotherapy, siRNA was used to deplete BRD4 in primary OS cells (494H, 148I, OS17). Consistent with the effects of combining JQ1 with doxorubicin, BRD4 depletion significantly increased the antiproliferative response of 494H, 148I and OS17 cells to doxorubicin (Fig. 6b), suggesting JQ1 could enhance the sensitivity of OS cells to standard therapy.

We further examined the utility of JQ1 in combination therapy approaches. BRD4 recruits the P-TEFb complex to promoters including *FOSL1*^37^, resulting in changes in RNA polymerase II phosphorylation and gene expression^38^. Cyclin dependent kinase 9 (CDK9) forms part of the P-TEFb complex, which is potently targeted by the CDK inhibitors, flavopiridol and dinaciclib^39^. Knockdown of *Cdk9* resulted in a loss of OS cell viability (Fig. 6c,d) and OS cells were highly sensitive to both flavopiridol and dinaciclib with IC_50_ values between 0.2 μM-0.33 μM and 0.007 μM-0.03 μM respectively (Fig. 6e,f and Supplementary Fig. 5). Treatment for 24 hrs induced a significant increase in apoptotic Annexin-V positive cells (Fig. 6g). We thus hypothesised that a multi-targeting approach of BRD4 activity through co-treatment with JQ1 and CDK inhibitors would be highly efficacious in OS.

Combinations of JQ1 with flavopiridol and dinaciclib were tested using effector concentration ranges below a 60% inhibition response. Viability was measured and combinatorial synergistic interactions were tested using the Bliss-additivity model. Greatly enhanced cell death was achieved when JQ1 was combined with flavopiridol or dinaciclib, reaching up to 50% more than any drug alone (Fig. 6h). Strong synergism between JQ1/flavopiridol and JQ1/dinaciclib was observed, reaching up to 300% and 80% more inhibition than predicted to be additive respectively (Fig. 6i). To further explore the synergy observed with co-inhibition of BETi and CDKs, we tested whether knockdown of BRD4 would synergise with dinaciclib to kill OS cells. OS cells were transfected with siRNAs to *BRD4* or a non-targeting siRNA together with 25 nM dinaciclib and cell viability assessed. BRD4 knockdown combined with dinaciclib was moderately synergistic (15.64-15.06%), with BRD4 siRNA/dinaciclib treated cells being far greater inhibited (44-62%) than non-targeted cells treated with dinaciclib (Fig. 6j). The combination of JQ1 with dinaciclib resulted in a significant increase in the number of Annexin-V positive cells (Fig. 6k). The concurrent inhibition of BRD4 and CDKs is potently synergistic at inducing apoptosis of OS cells.

## Discussion

The introduction of chemotherapy to OS treatment protocols resulted in a significant improvement in the survival of patients with localised tumours^2^. However, survival rates have plateaued and improvements in survival have been a result of improved delivery of existing treatments rather than from the rational application of new targeted agents^2^,^40^. In this study we have used a panel of OS cultures to show a potent class effect of BETi on OS cells. BETi, predominantly via inhibition of BRD4, had potent antiproliferative activity against human and mouse OS irrespective of origin (primary vs metastatic site), subtype, p53 status and proliferation rate, as well as potent *in vivo* activity in an OS preclinical model. Despite the strong link between BETi activity and MYC suppression in multiple models^23^,^25^,^28^,^29^,^30^,^36^, we identified JQ1 anti-OS activity is MYC independent and was instead driven by *FOSL1* suppression, similar to effects in lung cancer^27^. In addition, the anti-OS activity of JQ1 was selective, inducing rapid apoptosis in all primary OS cells, while normal osteoblasts remained protected from pro-apoptotic effects. Importantly, proof-of-principle studies for a BETi combinatorial treatment approach to OS are demonstrated. JQ1 sensitised OS cells to doxorubicin, a chemotherapeutic agent used in routine OS treatment practices. Furthermore, combining JQ1 with CDK inhibitors resulted in synergistic killing of OS, identifying a novel rational combinatorial treatment with agents in clinical trials. At least four BETi are currently being assessed in Phase I clinical trials. These studies provide a strong rationale for the further testing of BETi either alone or in combination clinically in OS.

Downregulation of MYC has been reported as the mechanism mediating BETi activity in many tumour models^23^,^24^,^25^,^28^,^29^. Surprisingly, despite evidence implicating *MYC* as an oncogenic driver in OS^11^,^12^,^13^, MYC was not downregulated by JQ1 in our studies. Instead, we demonstrated *FOSL1* is downregulated and is a significant contributor to the effects of JQ1. Although FOSL1 has been shown to be an important regulator of the osteoblastic content of bone by promoting osteoblast differentiation^20^,^21^, this is the first time *FOSL1* has been implicated as a potential therapeutic target in OS. How suppression of FOSL1 transcription factor mediates the antiproliferative effects of BETi in OS remains to be determined. A closely related family member, FOS, is strongly associated with OS^18^, and redundancy between FOSL1 and FOS has been reported^19^. In lung cancer cells where BETi was shown to suppress *FOSL1*, a downregulated FOS gene signature was detected^27^. A gene signature common to these transcription factors may be important for OS survival.

The loss of viability from FOSL1 knockdown was less than observed with either JQ1 treatment or BRD4 knockdown suggesting, not unexpectedly, a multi-factorial action of BETi. Multiple targets are likely involved, as previous studies have demonstrated that over-expression of a single putative target such as *MYC* or *FOSL1* was insufficient to completely rescue cells from JQ1^25^,^41^. Indeed we observed that JQ1 exposure repressed *RUNX2*, which has been linked to OS^8^,^9^,^42^. JQ1 has also been shown to downregulate anti-apoptotic genes such as *BCL2*^24^. Loss of BCL-2 has been shown to sensitise OS cells to doxorubicin^43^. An underappreciated aspect of the effects of JQ1 may be the upregulation of genes, which may also contribute to BETi activity. Further, JQ1 also inhibits BRD2 and BRD3. Although BRD4 loss was the most potent in reducing viability, knockdown of *Brd2* and *Brd3* in mouse OS cells also led to decreased survival, suggesting that the antiproliferative effects of JQ1 may not be fully mediated via a single target in BRD4.

JQ1 activity in OS cell lines has recently been reported in two independent studies that utilised long established OS cell lines^41^,^44^. We have confirmed their findings that *RUNX2* is suppressed in response to JQ1 treatment, however several important differences are evident. Both groups reported that JQ1 treatment repressed MYC transcription in a subset of OS cell lines^41^,^44^. Interestingly, a loss of MYC protein was not evident or failed to be reported in the long established OS cells in response to JQ1^41^,^44^, in contrast to other tumour models where BETi activity was correlated with significant reductions in MYC protein^25^,^29^,^45^. Further, we demonstrated apoptosis was rapidly induced by JQ1 treatment in all primary OS cell cultures examined, whereas the prior studies observed apoptosis to be variable and limited in OS cell lines^41^,^44^. Why such contrasting findings? It perhaps involves the models used for preclinical testing. Our studies have deliberately made use of primary OS cultures which provide a closer representation of the disease by limiting potential changes from long term *in vitro* culture. Extended culture conditions may result in alternative BRD4 genomic localisation patterns, rendering cell lines differentially susceptible to treatments. The cell type specific effects mediated by JQ1 is proposed to be controlled by cell specific asymmetrical super-loading of BRD4 at enhancers of genes involved in cell-fate determination and oncogenic drivers^23^,^46^. JQ1 treatment leads to a preferential loss of BRD4 at these enhancers and subsequent transcriptional repression^23^,^46^. Genome-wide super-enhancer H3K27ac ChIP-Seq analysis could distinguish differences that segregated diffuse large B cell lymphoma cell lines from primary samples^23^. Indeed, cell specific BRD4 super-loading of oncogenic drivers in OS cells may explain the protection of normal osteoblasts from the pro-apoptotic effects of JQ1 treatment. The protection of normal osteoblasts from the pro-apoptotic OS activity of JQ1 provides compelling rational for clinical testing, and warrants investigation of OS BRD4 super-enhancers to potentially identify oncogenic susceptibilities.

One function of BRD4 is to recruit P-TEFb to gene promoters where it phosphorylates RNA polymerase II to induce transcriptional elongation^38^. We tested whether concurrent inhibition of P-TEFb would co-operate with JQ1 by further compromising BRD4 function. JQ1 combined with the CDK inhibitors dinaciclib or flavopiridol led to synergistic killing of OS *in vitro*. Synergy was observed with both agents suggesting the effects were not compound specific, rather driven by “on target” inhibition of CDKs. The molecular basis of the synergistic reaction remains to be determined. Knockdown of BRD4 with dinaciclib was able to phenocopy the synergistic effects of the combined pharmacological approach. Dinaciclib has been shown to bind and inhibit the activity of BET proteins, albeit to levels significantly less than CDKs^47^. As synergy was still achieved with knockdown of BRD4, the interactions between the drugs is not likely driven by direct inhibition of BRD4 by dinaciclib. The more potent effect of JQ1 compared to BRD4 knockdown when combined with dinaciclib may reflect contributions from BRD2/3.

The results offer promise for increasing the *in vivo* efficacy of dinaciclib. Dinaciclib showed *in vitro* activity in multiple cancer cell lines and was well tolerated in pre-clinical testing^48^,^49^. We observed that primary OS cells are extremely sensitive *in vitro* to dinaciclib, with IC_50_ values in the low nanomolar range. However *in vivo* testing of dinaciclib in OS xenografts showed limited activity^48^. Apart from CLL, phase II trials of dinaciclib in multiple malignancies have not yielded encouraging responses^50^. Several Phase I trials have commenced testing dinaciclib in combination with bortezomib, dexamethasone and the AKT inhibitor MK2206. The current findings provide rationale for the testing of dinaciclib and BETi combination treatment.

In addition to dinaciclib, JQ1 improved OS cell response to doxorubicin. We demonstrated that JQ1 and doxorubicin induces additive effects in reducing OS cell viability. Although a synergistic response was not achieved, the results indicate the addition of JQ1 could reduce the dose of doxorubicin required to achieve the same degree of cell death. Given doxorubicin treatment is associated with severe life threatening side effects^4^, strategies that can lower the dose of doxorubicin could have significant benefit to patients’ morbidity and treatment tolerability.

The ability of BETi to target different oncogenic pathways in different cancers suggests they will have wide ranging clinical applications. Our studies provide strong evidence that BET inhibitors represent a novel therapeutic strategy to treat OS either as monotherapy or in combination with current or novel therapies. We demonstrate that primary tumour derived cell cultures from murine OS models or human patient material are highly sensitive to BETi and undergo apoptosis as the primary antiproliferative response. This contrasts with work in long established cell cultures, where apoptosis was variable and cell cycle arrest and induction of senescence was more prominent. This study emphasises the importance of testing novel agents and defining their mechanism of action in models more closely representative of the disease as it occurs in patients.

## Methods

### Cell culture

Cells were maintained in αMEM supplemented with 10% FCS. Human cell lines were purchased from ATCC (no authentication performed). Primary human OS cultures were derived from tumours from xenograft models^31^,^32^ (material generously provided by Dr Peter Houghton, Nationwide Children’s Hospital, Ohio; no authentication performed). Normal human osteoblasts were derived from bone marrow aspirates from normal healthy individuals^51^ (no authentication performed). Primary mouse OS cultures were derived from tumours from fibroblastic or osteoblastic models of OS^33^,^34^ (no authentication performed). Primary osteoblast cells were isolated from C57BL6 mouse collagenase digested bones. Osteoblastic cells were purified by flow cytometry (FACSAria, BD Biosciences) as previously described^52^,^53^, or directly cultured following collagenase digestion. Flow cytometry antibodies are described in Supplementary Table S1.

### Compounds

(-)-JQ1 (Cayman Chemicals), I-BET151 (ChemieTek), I-BET762 (Millipore), Dinaciclib and Flavopiridol (Selleckchem) were purchased. (+)-JQ1 was purchased (Cayman Chemicals) or synthesised as previously described^26^. Doxorubicin was obtained from St Vincent’s Hospital Pharmacy (Melbourne, Australia).

### Cell viability and proliferation assays

Cell were seeded 24 h prior to drug treatment at 2500/well in 96 well plates. Compounds, diluted in DMSO, were applied (0.01 nM to 10 μM) for 48–72 h and viability quantified using CellTitre-Glo (Promega). Half maximal inhibitory (IC_50_) values were calculated using Prism 6.0e software. Proliferation rate was determined by counting cells on 4-5 consecutive days using a BioRad TC10 counter (Hercules, CA, USA).

### Synergy calculations

Synergy was calculated using the Bliss-Additivity model^54^. Experimental responses > 15% above the predicted combined response value were considered to indicate synergism^54^.

### Flow Cytometry

Cells were stained with Annexin V-APC (BD Biosciences) (1:20) and 7AAD (5 μg/ml) and apoptotic cells quantified by FACS (LSRFortessa, BD Biosciences) and analysed using FlowJo software (v8.8.7). Cell cycle was determined by staining with propidium iodide (40 mg/ml) and quantifying DNA content by FACS. Cell cycle distributions were analysed using FlowJo software.

### RNA extraction and Real-time PCR

QPCR analyses were carried out using primers described in Supplementary Table S2 using methods as previously described^33^,^34^.

### Genome wide expression analyses

RNA-Sequencing was conducted on an Illumina HiSeq 2000 with 100 bp paired-end reads. Reads were aligned to the mouse genome build mm9/NCBI37 using Casava 1.7 and Bowtie v0.12.2 mapping software, normalised using Voom linear modeling^55^ and transcript abundance measured as reads per kilobase of exon per million mapped reads (RPKM)^56^. Datasets are deposited in GEO (accession number GSE58916). Gene expression data from a publicly available human OS dataset (accession number GSE33382^57^) were normalised using R/bioconductor.

### siRNA knockdown

Cells were transfected with Dharmacon On-Target Plus siRNA pools (GE Healthcare Life Sciences) (20 nM) complexed with DharmaFECT 3 (GE Healthcare Life Sciences) for 72 h. Cells transfected with a non-targeting On-Target Plus siRNA pool served as controls. Drug treatments commenced 24 to 48 h after siRNA transfection.

### Western blotting

Antibodies (BRD4 Bethyl Laboratories; c-MYC [9E10] Abcam ab32; FOSL1 Abcam ab65051; Cleaved caspase 3 Asp175 [5A1E] Cell Signaling Technologies 9664S; Pan Actin [Ab5] Thermo Scientific MS-1295-PO) were used at 1:1000 (FOSL1, Cleaved caspase-3), 1:10,000 (BRD4) and 1:3000 (pan-actin).

### Chromatin Immunoprecipitation

ChIP was carried out on 5 × 10^6^ cells as described previously^58^. Cells were treated with 1 μM JQ1 or 0.1% v/v DMSO for 24 hrs. Cells were fixed with 1% formaldehyde diluted in PBS for 10 min at room temperature. Cross-linking was quenched by incubating cells with 0.125 M glycine diluted in PBS for 10 min at room temperature. Cells were washed twice in PBS before being scraped from plates and pelleted by centrifugation at 400 x g for 5 minutes at room temperature. Cell pellets were diluted in sonication buffer (1% SDS, 10 mM EDTA, 50 mM Tris-HCl pH8.1) with cocktail protease inhibitors (Roche) and the DNA sheared to lengths between 100 and 400 bp using a UCD-200 Bioruptor (Diagnenode) on high for a total shearing time of 15 min (90 min of 10 s on and 50 s off), maintained at 4 degrees celcius during shearing. Cell debris was cleared by centrifugation for 10 min at 13,000 rpm at 4 degrees celcius and supernatants diluted 10-fold in ChIP dilution buffer (0.01% SDS, 1.1% Triton X-100, 1.2 mM ETA, 16.7 mM Tris-HCl pH 8.1, 167 mM NaCL) with protease inhibitors. After removing 20 μl of each sample as an input control, protein-DNA fragments were immunoprecipitated with 5 μg BRD4 antibody (Bethyl Laboratories) or control rabbit IgG (Merck Millipore), or no antibody overnight at 4 degrees celcius with rotation. Complexes were collected for 1.5 hrs at 4 degrees celcius with rotation with 60 μl of protein A dynabeads (Invitrogen) that had been pre-blocked for 1 hr in 0.5% BSA in TE buffer. Beads were washed one time each with Low Salt buffer (0.1% SDS, 1% Triton X-100, 2 mM EDTA, 20 m Tris-HCl pH8.1, 150 mM NaCl), High Salt buffer (0.1% SDS, 1% Triton X-100, 2 mM EDTA, 20 mM Tris-HCl pH8.1, 500 mM NaCl) and LiCl buffer (0.25 M LiCl, 1% NP40, 1% deoxycholate, 1 mM EDTA, 10 mM Tris-HCl pH8.1), followed by two washes with TE buffer. Protein-DNA complexes were eluted from the beads (0.1 M NaHCO_3_, 1% SDS) with 2 hrs incubation at 62 degrees celcius with shaking. Cross-links were reversed overnight at 65 degrees celcius with the addition of 0.2 M NaCl. Protein was digested by incubation with 50 μg of proteinase K at 45 degrees celcius for 1 hr. DNA was purified by phenol:chloroform:isoamyl alcohol (25:24:1) (Sigma) extraction and ethanol precipitation with GlycoBlue (Life Technologies) as a carrier. BRD4 bound and input samples were analysed by QPCR using primers that amplified regions of the *FOSL1*, *c-MYC* and *RUNX2* loci or a negative control background region (region devoid of genes) (sequences available in Supplementary table S2). Relative enrichment was determined by the 2^(−deltaCT)^ method normalizing to input controls and background regions.

### *In vivo* studies

All animal experiments were undertaken in accordance with the Australian Code of Practice for the Care and Use of Animals for Scientific Purposes (7th edition, 2004 NHMRC; the “approved guidelines”) and under experimental protocols approved by the St Vincent’s Hospital Melbourne Animal Ethics Committee. 494H mouse fibroblastic OS cells infected with pMMP-LucNeo virus were used to generate the model. 25,000 tumour cells (increased to 75,000 in a second independent study) resuspended in extracellular matrix (Cultrex PathClear BME Reduced Growth Factor Basement Membrane Extract, Trevigen) were implanted sc on the dorsal hind flank of 6–7 week old Balb/c nu/nu mice. Tumours were allowed to establish for 1 week and were visualised and quantitated by bioluminescence imaging before treatment commenced with JQ1 (50 mg/kg) or vehicle (10% DMSO in 10% hydroxypropyl beta cyclodextrin) daily by ip injection. Tumour volume was measured using an IVIS Spectrum Imaging System (Perkin Elmer). Mice were injected with XenoLight D-luciferin (15 mg/ml) (Perkin Elmer) intraperitoneally. Bioluminescence was quantitated using Living Images software (Perkin Elmer).

### Microcomputed tomography

Tumours were scanned *ex vivo* using a SkyScan 1076 scanner (Bruker). Data acquisition setting were: 18 μm voxel resolution, 0.5 mm aluminum filter, 50 kV voltage, 200 μA current, 2600 ms exposure, rotation 0.6**°** across 180**°,** and frame averaging of 1. Reconstruction was performed using NRecon (v.1.6.3.1). Analysis was performed using CT Analyser (CTAn, v.1.12.0.0).

## Additional Information

**How to cite this article**: Baker, E. K. *et al.* BET inhibitors induce apoptosis through a MYC independent mechanism and synergise with CDK inhibitors to kill osteosarcoma cells. *Sci. Rep*. **5**, 10120; doi: 10.1038/srep10120 (2015).

## Supplementary Material

Supplementary Information
