# Supplementary material for: BET inhibitors induce apoptosis through a MYC independent mechanism and synergise with CDK inhibitors to kill osteosarcoma cells
Source: Sci Rep. 2015 May 6;5:10120. doi: 10.1038/srep10120 (PMC4421868; doi:10.1038/srep10120)
Supplement: Supplementary Information [file srep10120-s1.pdf]

Supplementary Information:

BET inhibitors induce apoptosis through a MYC independent mechanism and synergise with CDK inhibitors to kill osteosarcoma cells

Emma K Baker[1,2,*], Scott Taylor[1], Ankita Gupte[1], Phillip P Sharp[3], Mannu Walia[1], Nicole C Walsh[1,2], Andrew CW Zannettino[4], Alistair M Chalk[1,2], Christopher J Burns[3,5] and Carl R Walkley[1,2,*].

Supplementary Table S1: Antibodies used for flow cytometry

Antibodies	Clone	Conjugate	Supplier
Sca-1	D7	PacBlue	Biolegend
CD51	RMV-7	PE	BD Biosciences
CD31	390	APC	eBioscience
Lineage			
CD2	RM2-5	Biotin	eBioscience
CD3e	145-2C11	Biotin	eBioscience
CD4	GK1-5	Biotin	eBioscience
CD5	53-7.3	Biotin	eBioscience
CD8a	53-6.7	Biotin	eBioscience
B220	RA3-6B2	Biotin	eBioscience
Gr-1	RB6-8C5	Biotin	eBioscience
CD11b	M1/70	Biotin	eBioscience
Ter-119	TER-119	Biotin	eBioscience
CD45	30-F11	Biotin	eBioscience
Streptavidin		Qdot-605	Life Technologies

Supplementary Table S2: Primer Sequences

QPCR Analysis	Species	Primer	Forward Sequence	Reverse Sequence	Reference
Gene Expression	Human	*BRD2*	5'-GGG GTG GCA GTG CTG CTT TA-3'	5'-GCT CAG CTG CCG CTT CTC AT-3'	
	Human	*BRD3*	5'-ACT CCA ACC CCG ACG AGA TA-3'	5'- TCT TCC CGC TTG CTG AGA AC-3'	
	Human	*BRD4*	5'-GGA GCC ATC TCT GTT TCG GA-3'	5'-TAG GCA GGA CCT GTT TCG GA-3'	
	Human	*BRD4*	5'-GTT GAT GTG ATT GCC GGC TC-3'	5'-GGA GCC ATC TCT GTT TCG GA-3'	
	Human	*FOS*	5'-CCA AGC GGA GAC AGA CCA AC-3'	5'-ATC AGG GAT CTT GCA GGC AG-3'	
	Human	*FOSL1*	5'-CTGCAGGCGGAGACTGACAA-3'	5'-TCCGGGATTTTGCAGATGGG-3'	
	Human	*MYC*	5'-AGG GAT CGC GCT GAG TAT AA-3'	5'-TGC CTC TCG CTG GAA TTA CT-3'	1
	Human	*HAS2*	5'-AATTTTGGAAACTGCCCGCC-3'	5'-TCACAATGCATCTTGTTCAGCTC-3'	
	Human	*RUNX2*	5'-CCT AAA TCA CTG AGG CGG TC-3'	5'-CAG TAG ATG GAC CTC GGG AA-3'	
	Human	*PGK1*	5'-CATACCTGCTGGCTGGATGG-3'	5'-CCCACAGGACCATTCCACAC-3'	
	Mouse	*Brd2*	5'-GCT GAG CGG CGG CGG TTC CC-3'	5'-GTA AAG CTG GTA CAG AAG CC-3'	
	Mouse	*Brd3*	5'-GGA CTC AAA CCC AGA CGA GAT T-3'	5'-TGT TGA CAA TGG TTT CCT CTG C-3'	
	Mouse	*Brd4*	5'-CCA TGG ACA TGA GCA CAA TC-3'	5'-TGG AGA ACA TCA ATC GGA CA-3'	4
	Mouse	*Fos*	5'-TAC TAC CAT TCC CCA GCC GA-3'	5'-GCG CAA AAG TCC TGT GTG TT-3'	
	Mouse	*Fosl1*	5'-GTA CCG AGA CTA CGG GGA AC-3'	5'-ACA AGG TGG AAC TTC TGC TG-3'	
	Mouse	*Myc*	5'-AGG AGA CAC CGC CCA CCA CC-3'	5'-TGC TGT GGC CTC GGG ATG GA-3'	
	Mouse	*Has2*	5'- GGC CGG TCG TCT CAA ATT CA-3'	5'-ACA ATG CAT CTT GTT CAG CTC-3'	
	Mouse	*Runx2*	5'-CTCCGCTGTTATGAAAAACC-3'	5'-TGAAACTCTTGCCTCGTCC-3'	
	Mouse	*Hprt*	5'-TGATTAGCGATGATGAACCAG-3'	5'-AGAGGGCCACAATGTGATG-3'	
	Mouse	*Cdk9*	5'-AGG TGG CTC TGA AGA AAG TGT-3'	5'-TAT ACG GTG AGG CTT TGG TCC-3'	
ChIP	Human	*FOSL1* coding	5'-CAT TGC AGT GGT TCC G-3'	5'-CCC TCC TAA GCC TGT GCT CTC-3'	3
	Human	*FOSL1* promoter	5'-TGT ATG GGC AGC TAC GTC AGG-3'	5'-GCC TCC CCA AGT CCG-3'	3
	Human	*FOSL1* enhancer	5'-GGT GCC CAT TTC CTG TCG-3'	5'-GAC TCG GCG GAA CGG-3'	3
	Human	*RUNX2* coding	5'-GAC GAG CTG AGA TCG GTC AG-3'	5'-AAT CAA CTT CCA GTG CCG GT-3'	2
	Human	*RUNX2* promoter	5'-TGA GCG GGG AGT AGA AAG GA-3'	5'-ACA AAT GCT GAA GGA GCC CA-3'	
	Human	*c-MYC* TSS (1)	5'-ACA CTA ACA TCC CAC GCT CTG-3'	5'-GAT CAA GAG TCC CAG GGA GA-3'	1
	Human	*c-MYC* TSS (2)	5'-GGT CGG ACA TTC CTG CTT TA-3'	5'-GAT ATG CGG TCC CTA CTC CA-3'	1
	Human	Null region	5'-TCC TGG GTA GGA ACC AGT TG-3'	5'-ACT CAC CAA GAG CTC CTC CA-3'	1

References:

1 Delmore, J.E. *et al.* BET bromodomain inhibition as a therapeutic strategy to target c-Myc. *Cell* **146,** 904-917 (2011).

2 Lamoureux, F. *et al.* Selective inhibition of BET bromodomain epigenetic signalling interferes with the bone-associated tumour vicious cycle. *Nature Communications* **5,** 3511 (2014).

3 Zippo, A. *et al.* Histone crosstalk between H3S10ph and H4K16ac generates a histone code that mediates transcription elongation. *Cell* **138:** 1122-1136 (2009).

4 Zuber, J. *et al*. RNAi screen identifies Brd4 as a therapeutic target in acute myeloid leukaemia. *Nature* **478,** 524-528 (2011).

5 Kuijjer, M.L. *et al.* Identification of osteosarcoma driver genes by integrative analysis of copy number and gene expression data. *Genes Chromosomes Cancer* **51(7),** 696-706 (2012).

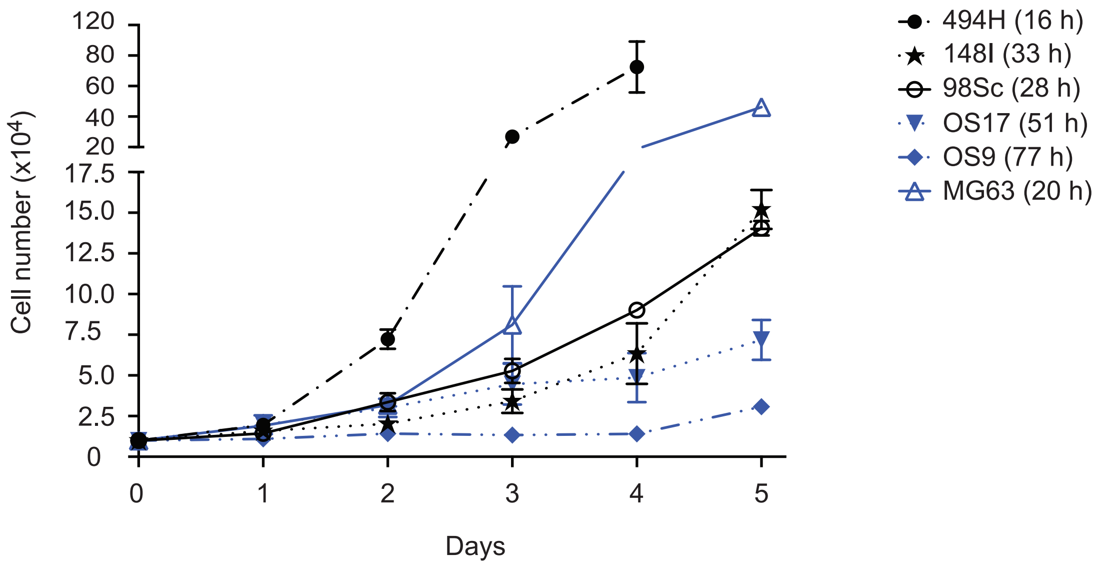

Supplementary Figure 1. The basal proliferation rate of OS cells does not impact on sensitivity to JQ1. Equal numbers of mouse (black) and human (blue) OS cells were seeded on day 0 (10,000 per well) and cell numbers counted every day for four to five days, depending on when confluency was reached. Data is represented as mean cell number ±SEM (n=2). The mean doubling time of each cell culture was calculated from two independent experiments and is represented in brackets.

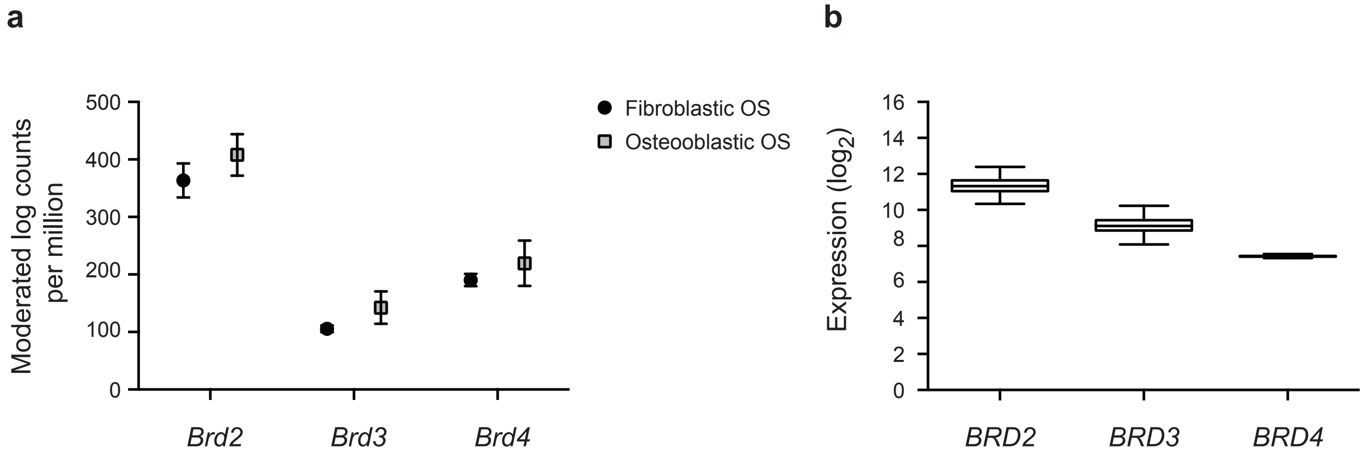

Supplementary Figure 2. JQ1 targets, *BRD2*, *BRD3*, and *BRD4*, are expressed in human and mouse OS cells. (a) Expression levels (moderated log counts per million) for *Brd2*, *Brd3*, and *Brd4* were assessed in an RNA-Seq dataset from mouse fibroblastic OS tumours (n=3) and mouse osteoblastic OS tumours (n=3). The data represents mean counts ±SEM. (b) Expression levels (log_2 transformed) for *BRD2*, *BRD3*, and *BRD4* were assessed in a publicly available microarray dataset from human OS samples (GSE33382[5]). The boxplots show the minimum and maximum log_2 values as whiskers, the centre line as the median, and the upper and lower boundaries as the 75th and 25th quartiles respectively.

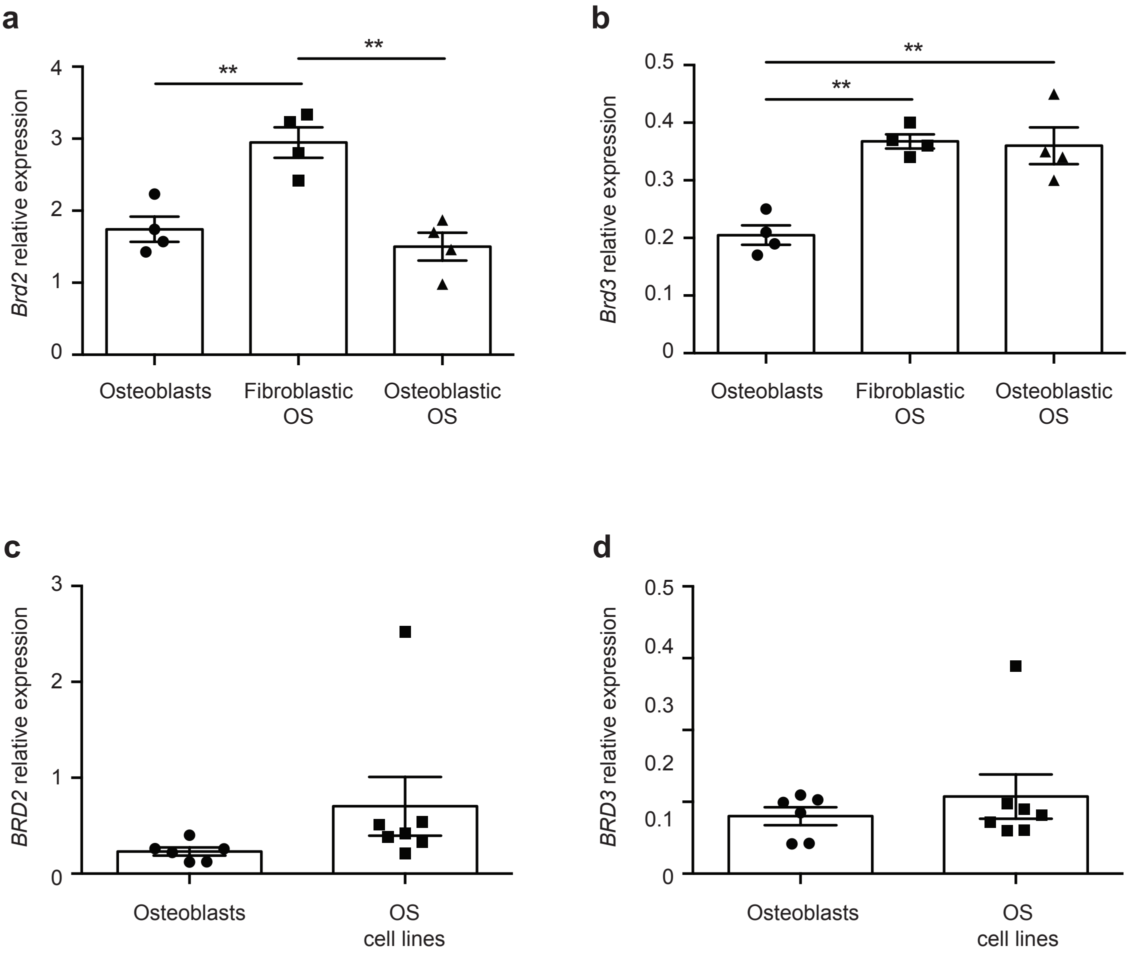

Supplementary Figure 3. Expression levels of *Brd2* and *Brd3* in mouse and human OS cells compared to normal osteoblastic cells. (a-b) Expression levels of *Brd2* and *Brd3* were assessed in mouse primary fibroblastic OS (494H, 493H, 716H, 202V), mouse primary osteoblastic OS (148I, 98Sc, 89R, 147H) cells compared to normal osteoblast cells (n=4 independently derived samples) by QPCR. . Expression levels were normalised to *Hprt*. Mean relative expression ±SEM, ** p<0.01 ANOVA Tukey multiple comparisons test. (c-d) Expression levels of *BRD2* and *BRD3* were assessed in human OS cells (U2OS, SJSA-1, B143, MG63, SAOS-2, G292, OS17) compared to normal osteoblast cells (n=6 independently derived samples) using QPCR. Expression levels were normalised to *PGK1*. Mean relative expression ±SEM.

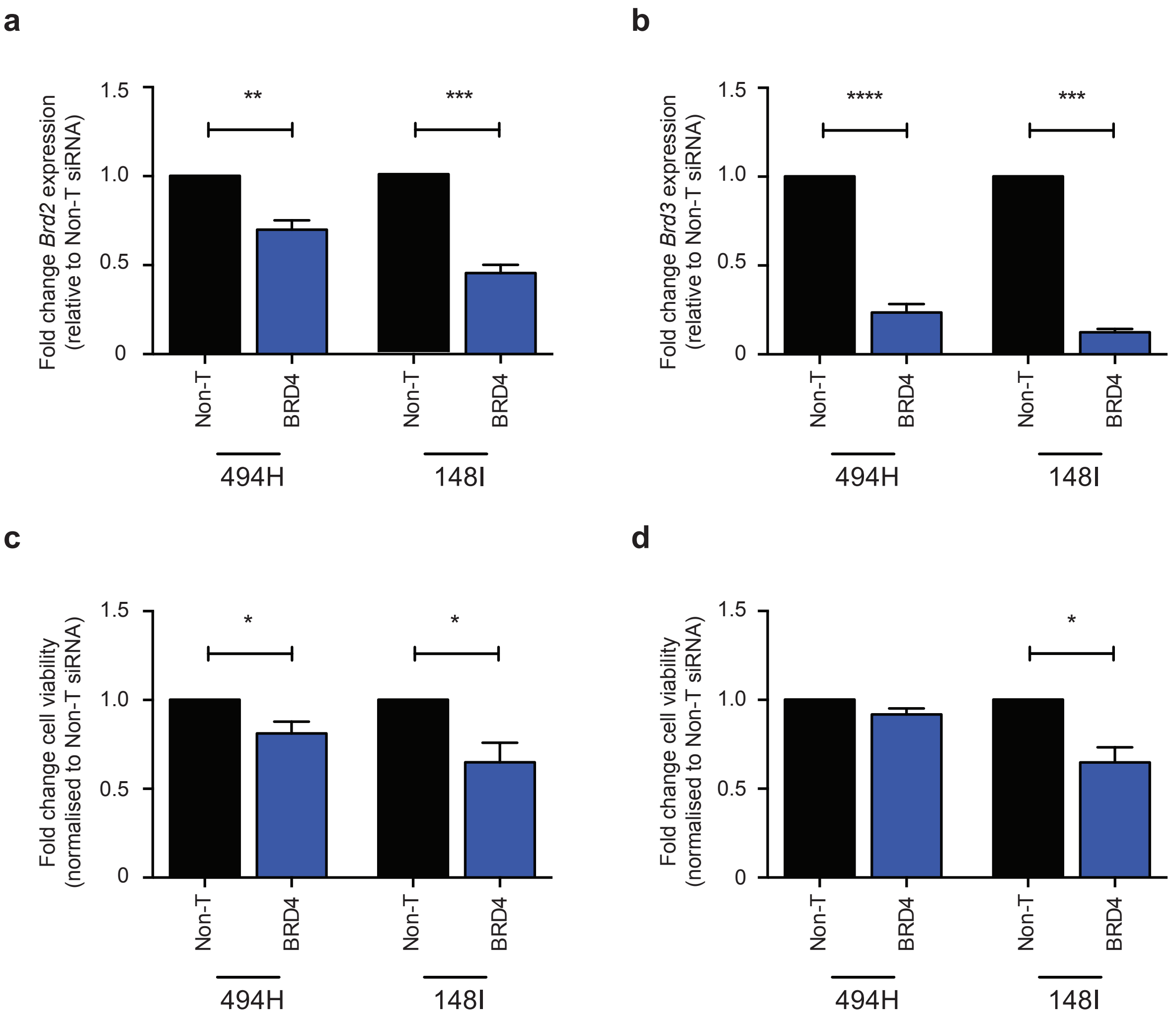

Supplementary Figure 4. Knockdown of *Brd2* and *Brd3* only have a moderate impact on OS cell viability. Mouse OS cells (494H, 148I) were transfected with siRNA smart pools against *Brd2* and *Brd3* and compared to cells transfected with a smart pool of non-targeting siRNAs (Non-T). Cells were transfected for 72 hrs and effects on *Brd2* and *Brd3* mRNA expression and cell viability assessed. (a) *Brd2* mRNA levels were assessed by QPCR. Mean fold change *Brd2* expression ±SEM (n=3). (b) *Brd3* mRNA levels were assessed by QPCR. Mean fold change *Brd3* expression ±SEM (n=3). (c) *Brd2* knockdown reduces OS cell viability. Data represents mean number of viable cells normalised to Non-T siRNA ±SEM (n=3). (d) *Brd3* knockdown reduces OS cell viability. Data represents mean number of viable cells normalised to Non-T siRNA ±SEM (n=3). *p<0.05, **p<0.01, ***p<0.001, ****p<0.0001 student's t test.

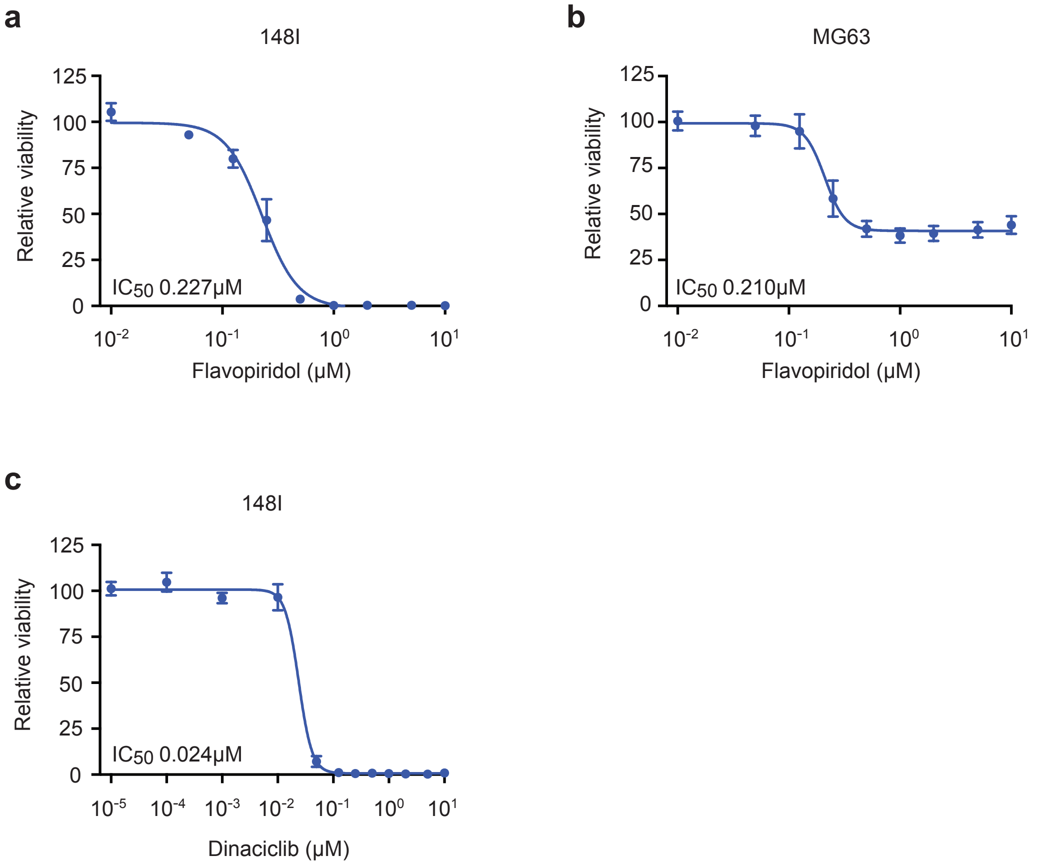

Supplementary Figure 5. OS cells are sensitive to CDK inhibitors. Dose response curves of mouse 148I and human MG63 OS cell cultures treated for 48 h with the CDK inhibitors flavopiridol (a-b) and dinaciclib (c). Cell viability was measured by quantification of ATP levels (CellTitre-Glo, Promega). Data represents mean cell viability ±SEM, (n=2) with average IC_{50} values.
